# Supplementary material for: Investigating the factors influencing antibiotic use practices and their association with antimicrobial resistance awareness among poultry farmers in Enugu State, Nigeria
Source: bioRxiv. 2025 Feb 10:2025.02.08.637249. Preprint. [Version 1] doi: 10.1101/2025.02.08.637249 (PMC11844400; doi:10.1101/2025.02.08.637249)
Supplement: Supplement 1 [file media-1.pdf]

Supplementary Information

Investigating the factors influencing antibiotic use practices and their association with antimicrobial resistance awareness among poultry farmers in Enugu State, Nigeria

Chika P. Ejikeugwu[1,2]*, Emmanuel A. Nwakaeze[3], Chikaodi W. Aniekwe[1], Euslar N. Onu[4], Michael U. Adikwu[5], Peter M. Eze[6,7]

[1]Department of Pharmaceutical Microbiology and Microbiology, Enugu State University of Science and Technology (ESUT), Agbani, Nigeria

[2]Department Angewandte Mikrobielle Ökologie, Helmholtz-Zentrum für Umweltforschung – UFZ, Leipzig, Germany

[3]Department of Pharmaceutical Microbiology and Microbiology, Chukwuemeka Odumegwu Ojukwu University, Igbariam, Nigeria

[4]Department of Microbiology, Faculty of Biological Science, Alex Ekwueme Federal University Ndufu-Alike, Ikwo, Nigeria

[5]Department of Pharmaceutics, University of Nigeria Nsukka, Enugu, Nigeria

[6] Department of Environmental Health Science, Nnamdi Azikiwe University, Awka, Nigeria

[7] School of Biological Sciences, Queens University Belfast, Northern Ireland, United Kingdom

***Corresponding author:** Chika P. Ejikeugwu (chika.ejikeugwu@esut.edu.ng)

Table of content

Table S1: Relationship between respondents' knowledge about antibiotic use and their socio-demographic data **3**

Table S2: Knowledge, Attitude and Practice on Antibiotics Use **4**

Table S3: Knowledge, Attitude and Practice on Antimicrobial Resistance (AMR) **6**

Table S4: Relationship between respondents' knowledge about AMR and their socio-demographic data **7**

Table S1: Relationship between respondents' knowledge about antibiotic use and their socio-demographic data

**Demographic variable**		Knowledge level				**Total**		**p-value** (calculated using 2-way ANOVA)	Remark
		**No**		**Yes**					
		n	%	n	%	n	%		
**Gender**	Male	6	6.82	82	93.18	88	100	0.570	p>0.05 (There is no significant difference in the knowledge level of the male and female respondents).
	Female	3	2.68	109	97.32	112	100		
**Age**	<20 years	0	0	8	100	8	100	0.392	p>0.05 (There is no significant difference in the knowledge level of respondents in the different age groups).
	21 – 30 years	3	5.56	51	94.44	54	100		
	31 – 40 years	1	2	49	98	50	100		
	41 – 50 years	4	9.09	40	90.91	44	100		
	51-60 years	0	0	21	100	21	100		
	>60 years	1	4.35	22	95.65	23	100		
**Marital Status**	Single	6	9.09	60	90.91	66	100	0.487	p>0.05 (There is no significant difference in the knowledge level of respondents with different marital statuses).
	Married	2	1.52	130	98.48	132	100		
	Divorced	0	0	1	100	1	100		
**Occupational Status**	Employed	2	2.70	72	97.30	74	100	0.448	P>0.05 (There is no significant difference in the knowledge level of respondents with different occupational statuses).
	Self Employed	1	2.38	41	97.62	42	100		
	Housewife	0	0	16	100	16	100		
	Househusband	0	0	1	100	1	100		
	Student	3	8.33	33	91.67	36	100		
	Retired	1	6.25	15	93.75	16	100		
	Others	1	7.14	13	92.86	14	100		
**Highest Academic Qualifications**	Primary school	1	20	4	80	5	100	0.457	P>0.05 (There is no significant difference in the knowledge level of respondents with different academic qualifications).
	Secondary school	1	1.61	61	98.39	62	100		
	University	3	3.95	73	96.05	76	100		
	Diploma	2	16.67	10	83.33	12	100		
	OND/HND	0	0	11	100	11	100		
	Masters	1	5.56	17	94.44	18	100		
	Doctorate (PhD)	0	0	8	100	8	100		
**Internet Usage**	Everyday	4	3.70	104	96.30	108	100	0.467	P>0.05 (There is no significant difference in the knowledge level of respondents with varying degrees of internet use).
	Often	1	1.75	56	98.25	57	100		
	Rarely	2	9.52	19	90.48	21	100		
	Never	1	7.69	12	92.31	13	100		

Table S2: Knowledge, Attitude and Practice on Antibiotics Use

Variable	Response	n	%
Did you administer antibiotics to your birds within the past year?	Yes	181	90.5
	No	19	9.5
Where did you obtain the antibiotics that you have administered within the past year?	Patent chemist shop	29	14.5
	Pharmacy	46	23
	Open market	42	21
	Drug vendors	12	6
	Hospital	37	18.5
	Veterinary clinic	140	70
Do you consider cost, quality and the intended usage when buying antibiotics for your birds?	Yes	192	96
	No	4	2
	No idea	2	1
	No response	2	1
What was your reason for administering antibiotics to your birds?	For growth promotion	25	12.5
	For prophylaxis	87	43.5
	For treating infection	161	80.5
	For feed enhancement	10	5
	Other	3	1.5
When do you start the use of antibiotics?	When there are no symptoms of infection	48	24
	When there are symptoms of infection	154	77
	During breeding	76	38
	Upon the recommendation of a veterinarian	30	15
	At all times	5	2.5
	When I feel like	4	2
Did you know the class, names or brands of the antibiotics you administered to your birds	Yes	160	80
	No	40	20
List of the antibiotics class or names	Gentamicin	134	67
	Amoxicillin	118	59
	Doxycycline	142	71
	Ciprofloxacin	143	71.5
	Septrin (co-trimoxazole)	20	10
	Ampicillin	150	75
	Tetracycline	100	50
	No response	41	20.5
Antibiotics are good for promoting the growth of poultry birds	Correct	178	89
	Wrong	5	2.5
	I don’t know	14	7
	No response	3	1.5
Unnecessary use of antibiotics leads to that drug losing its effectiveness in future	Yes	165	82.5
	No	7	3.5
	I don’t know	24	12
	No response	4	2
How frequent do you administer antibiotics?	Daily	10	5
	Weekly	24	12
	Bi-weekly	59	29.5
	Monthly	96	48
	Yearly	4	2
	No response	7	3.5
Do you know the amount or dosage of antibiotics administered?	Yes	188	94
	No	5	2.5
	No response	7	3.5
Poultry farmers should stop introducing antibiotics in feeds?	Agree	30	15
	Disagree	144	72
	I don’t know	21	10.5
	No response	5	2.5

Do you give your poultry birds more than one type of antibiotics?	Yes	172	86
	No	16	8
	I don't know	5	2.5
	No response	7	3.5
Do you seek for the services/opinion of an expert before deciding to use antibiotics?	Yes	173	86.5
	No	19	9.5
	No response	8	4
Do you agree that antibiotics should not be used in poultry farms?	Yes	24	12
	No	149	74.5
	I don't know	22	11
	No response	5	2.5
Do you agree that there are better alternatives (e.g., vaccination) to the use of antibiotics?	Yes	84	42
	No	10	5
	I don't know	101	50.5
	No response	5	2.5
Do you have access to veterinary services?	Yes	185	92.5
	No	10	5
	No response	5	2.5

Table S3: Knowledge, Attitude and Practice on Antimicrobial Resistance (AMR)

Variable	Response	n	%
Have you heard of antimicrobial resistance (AMR)?	Yes	66	33
	No	130	65
	No response	4	2
The use of antibiotics in poultry farm promotes AMR	Strongly Agree	10	5
	Agree	85	42.5
	Disagree	45	22.5
	Strongly disagree	7	3.5
	I don't know	50	25
	No response	3	1.5
AMR is threat to humans, animals and the general environment	Strongly Agree	36	18
	Agree	111	55.5
	Disagree	4	2
	Strongly disagree	2	1
	I don't know	44	22
	No response	3	1.5
How interested are you to learn about AMR?	Interested	173	86.5
	Not interested	6	3
	Undecided	18	9
	No response	3	1.5
Are you committed to stop AMR spread by ending the use of antibiotics in poultry feed?	Yes	79	39.5
	No	42	21
	I don't know	76	38
	No response	3	1.5
What do you think could cause AMR? Please select or tick all that you think applies	Unnecessary antibiotic use	78	39
	Excessive antibiotic use	163	81.5
	Lack of personal hygiene (e.g., hand washing)	33	16.5
	Stoppage of antibiotics during treatment	45	22.5
	Over the counter availability and use of antibiotics	17	8.5
	Using antibiotics without prescription	28	14
	Others	16	8
	I don't know	21	10.5
Are you worried about the impact AMR will have on your health and that of others?	Yes	107	53.5
	No	46	23
	I don't know	44	22
	No response	3	1.5
Are you at risk of getting an AMR infection?	Yes	32	16
	No	46	23
	I don't know	119	59.5
	No response	3	1.5

Table S4: Relationship between respondents' knowledge about AMR and their socio-demographic data

**Demographic variable**		Knowledge level **No** n	%	**Yes** n	%	**Total** n	%	**p-value** (calculated using 2-way ANOVA)	Remark
**Gender**	Male	43	48.86	45	51.14	88	100	0.410	p>0.05 (There is no significant difference in the knowledge level of the male and female respondents).
	Female	64	57.14	48	42.86	112	100		
**Age**	<20 years	4	50	4	50	8	100	0.005	P≤0.05 (There is significant difference in the knowledge level of respondents in the different age groups).
	21 – 30 years	25	46.30	29	53.70	54	100		
	31 – 40 years	28	56	22	44	50	100		
	41 – 50 years	24	54.55	20	45.45	44	100		
	51-60 years	10	47.62	11	52.38	21	100		
	>60 years	16	69.57	7	30.43	23	100		
**Marital Status**	Single	27	40.91	39	59.09	66	100	0.082	p>0.05 (There is no significant difference in the knowledge level of respondents with different marital statuses).
	Married	79	59.85	53	40.15	132	100		
	Divorced	0	0	1	100	1	100		
**Occupational Status**	Employed	31	41.89	43	58.11	74	100	0.006	P≤0.05 (There is significant difference in the knowledge level of respondents with different occupational statuses).
	Self Employed	23	54.76	19	45.24	42	100		
	Housewife	13	81.25	3	18.75	16	100		
	Househusband	0	0	1	100	1	100		
	Student	17	47.22	19	52.78	36	100		
	Retired	11	68.75	5	31.25	16	100		
	Others	11	78.57	3	21.43	14	100		
**Highest Academic Qualification**	Primary school	5	100	0	0	5	100	0.020	P≤0.05 (There is significant difference in the knowledge level of respondents with different academic qualifications).
	Secondary school	43	69.35	19	30.65	62	100		
	University	37	48.68	39	51.32	76	100		
	Diploma	5	41.67	7	58.33	12	100		
	OND/HND	7	63.64	4	36.36	11	100		
	Masters	3	16.67	15	83.33	18	100		
	Doctorate (PhD)	1	12.5	7	87.5	8	100		
**Internet Usage**	Everyday	51	47.22	57	52.78	108	100	0.014	P≤0.05 (There is significant difference in the knowledge level of respondents with varying degrees of internet use).
	Often	27	47.37	30	52.63	57	100		
	Rarely	16	76.19	5	23.81	21	100		
	Never	12	92.31	1	7.69	13	100		
